# Case report: Suicide and inhibitions of thinking—An integrative view from traditional psychoanalytic and mentalizing perspectives

**DOI:** 10.3389/fpsyt.2023.1170924

**Published:** 2023-05-09

**Authors:** Chong Seng Choi

**Affiliations:** Department of Psychiatry, Faculty of Medicine and Health Sciences, University Putra Malaysia, Serdang, Selangor, Malaysia

**Keywords:** suicide, inhibition of thinking, core conflict, dysfunctional mental processing, psychoanalysis, mentalizing

## Abstract

Suicide has been the subject of exploration in psychoanalysis. From Freud's internalized aggression and self-objectification in melancholic depression to contributions from object relation and self-psychology theorists, several of these central clinical concepts seem to share the commonality that one encounters an inhibition of thinking in a suicidal state of mind. Their freedom of thought is inhibited unswervingly despite the notion that we are born to think. Most psychopathologies, including suicide, relate to how we are often stuck with our thoughts. Thinking beyond this sense comes with significant emotional resistance. This case report follows through an attempt to integrate the hypothesized inhibitions on one's capability to think, involving one's own core conflicts and dysfunctional mental processing from the traditional psychoanalytic and mentalizing perspectives. The author hopes that further conceptualizations and research will empirically investigate these assumptions, potentially improving suicide risk assessment and prevention and enhancing psychotherapeutic outcomes.

## 1. Introduction

Psychoanalysis, as originated in the clinical observations by Freud, is a metapsychology of how the mind works and a psychotherapeutic method for psychic problems. Both of these applications have roles in understanding and helping people with suicidal behavior. Bion, one of the prominent psychoanalytic thinkers, had proposed the principles of mental functioning, in which inhibitions of thinking results from the inability to tolerate frustration. A capacity for thinking is developed to come to terms with frustration intrinsic to our appreciation of the gap between a wish and its fulfillment (1). The centrality of a traditional psychoanalytic understanding of suicide phenomena is the person's internal subjective experience of increasingly unbearable psychic pain and the urgent need for relief from intense emotions, such as shame, humiliation, and rage (2), leading to impairment of reality testing and judgment. These have a destructive nature; as Stekel (1910) said, “*No one kills himself who has never wanted to kill another or at least wished the death of another*,” which becomes the foundation for all subsequent psychoanalytic thinking on suicide (3).

Within the classical psychoanalytic theoretical construct, mental representation is used to understand and explain one's inner world (4, 5). People who are unable to integrate conflictual mental representations tend to struggle with core conflicts. An infant is said to form mental representations with symbolic content (6) and possesses primitive thoughts at the preverbal level (7). The formation of conflicting mental representations involves omnipotent phantasies of a need-satisfying object (7), much driven by instincts and early wishes. However, the gap between the experience of the need and its satisfaction creates frustration that makes thinking possible and emanates (1). In her paper “Psychoanalysis and Freedom of Thought” (1977), Segal wrote that the first step in thinking is by forming a conception of the actual circumstances and endeavoring to make a fundamental alteration in them (7). When one starts to think about their wishes and fantasies, they begin to be recognized as one's mind. This process moves into the realm of thinking, subjecting the thoughts to the possibility that they can be different from others and the harsh external reality. In this sense, the core conflicts are dealt with thorough thinking.

Meanwhile, mentalizing is a mental process involving imaginative mental activity to understand others and oneself, namely perceiving and interpreting human behavior in terms of intentional mental states (8). To be able to mentalize means thinking imaginatively and flexibly, ascribing meanings to human behavior, and referring to feelings, beliefs, desires, thoughts, and goals. Unfortunately, effective mentalizing is reduced in individuals who have experienced childhood adversity, possibly due to the impact of trauma on cognitive functioning. These lead to epistemic distrust and impaired affect regulation. Furthermore, the ability to reflect and understand the consequences of aggressive and self-destructive actions is hindered. This is as posited by Fonagy (1993), in which a boy's self-sabotaging behavior could be deadly; “his primitive reflective self did not see the death of his body as leading to the death of his mental self” (9).

I would like to highlight, with the help of a patient's case study and during one of the therapy sessions with her, the process of inhibitions of thinking, which leads to recurrent suicidal attempts, based on the hypothesized two core pathologies of having conflictual mental representations (i.e., the traditional psychoanalytic perspective) and dysfunctional mental processing (i.e., the mentalizing perspective).

## 2. Case presentation

A 35-year-old woman who had comorbid bipolar II and borderline personality disorders began her psychotherapy 3 years ago after transferring care from her resigned psychiatrist. In the past, there were a few unsuccessful medications to contain her emotional dysregulation, impulsivity, and suicidal behaviors. These included mood stabilizers such as lithium, sodium valproate, and even atypical antipsychotics. She often plugged into the depressive phase, thinking she was not achieving, unlike her siblings, and was highly self-critical. While she was in the hypomanic stage, it would be fused with her drive to improve her life.

Before we met, I had heard stories of her repeatedly arguing with the clinic nurse over some appointment matters. This narrative gave me the impression that she could be a *difficult patient*. I could remember the first time we saw each other; she was very wary as I was of how each other would be. During the discussion to lay out our treatment's direction, she wanted psychotherapy and wanted to continue her medications. Initially, I informed her that I could provide psychotherapy, but I would prefer her to see another psychiatrist whose role was primarily to take care of her medications. She found it very hard to understand the justifications for my suggestion. At that time, she must have experienced me as difficult to negotiate.

Previously, she had attended a course in cognitive behavior therapy, which she felt was not helpful. In her words, the treatment just wanted her to change and that was described as relatively rigid on how it set out to be. We currently work in the frame of psychoanalytically informed psychotherapy. She, too, found the weekly fixed schedules rigid. During our initial contact, she wanted psychotherapy to improve her relationships with others, especially with her boyfriend. She described him as immature, inexperienced, not educated, and stubborn. Despite how frequently she had tried to advise or guide him to change to be a better person for their future, she still failed to see any improvements. Instead, they were in a relationship filled with arguments whenever her expectations for him met with disappointments. However, she failed to acknowledge that he was there to support her emotionally and offer respite each time she had trouble with others.

In addition to her intimate relationship, the patient quickly entered arguments with others when she perceived them as crossing her boundaries or intimidating her. She constantly felt there was a need to speak up and defend herself. She attributed this defensive mode to the period when she had enough *taken in* people's defamation, which, while working for a corporate company, had led to her numerous mistreatments from her superiors and customers, eventually plugging her into depression. Since her resignation, she dared not to hold up any occupation that required her to come directly or frequently encounter people. She worried this could trigger her emotional instability and anger outbursts.

Part of her depressive symptoms was this incredible guilt feeling each time she lashed out her anger at others. These outbursts could lead her to repeatedly experience herself as being a bad person. As she became intolerable with these thoughts, she would commit multiple acts of self-harm and attempted suicide, such as cutting or overdosing on medications. The patient had countless attempts of such behavior, the last one was carried out when her boyfriend proposed a breakoff when she felt he was not taking their future seriously. Even though a minor life event triggered it, she lashed out her anger and frustration toward him and subsequently cut herself and ingested multiple pills of medications. She was then found by her boyfriend and was admitted to the hospital. Although her responses were impulsive and damaging, she believed she had thought about others' feelings enough. She often had to keep an eye on others' demeanor and analyze people's motives, as this became her mental default mode. She needed to make sure that they did not cross her boundary, and at the same time, she was constantly cautious of not offending anyone too. Her inner world is filled with a constant fear of despise by people because of her weaknesses and inadequacy.

She thought that she had inherited her father's stormy temperament. The difference was that he would never feel guilty after demonstrating his anger. She recalled growing up in a traditional Asian Chinese environment where her father emphasized a lot on achievements, was demanding, and allowed no room for emotional sharing. He was strict and fierce and would use physical punishment at home whenever he regarded his children were misbehaving. Her mother was even scolded by him for the children's wrongdoings. Thus, she was unhappy with her childhood, feeling that there was no one to protect her and that she needed to be hypervigilant at home. She had spent much time observing her father's reactions every moment. Even up to date, their relationship was strained. The experience, as she thought, had contributed to her sensitivity within the context of interpersonal contacts. She admitted that she could be easily provoked by others, picking up cues that they were stepping over her, and therefore, she needed to act very fast to protect herself. She also thought that her emotional instability was primarily contributed by those around her who were insensitive, mistreating her, and even looking down upon her.

## 3. Excerpt of a psychotherapy session

It was a rescheduled session as the patient had requested to change her previous appointment, informing me that she felt unwell. Before we started, I, too, noticed that she had been unusually late for her current session. The presence of these unfamiliar demeanors made me ponder on a hindrance to the progress of our therapeutic work. She started by telling me that she had attempted suicide again because of her anger and disappointment toward her boyfriend.

Therapist: I see you are frustrated, but I'm wondering if it's easy for him, too.

Patient: No, but he needs to improve.

Therapist: Let's stop and think about it for a moment. When you talked about hurting yourself, I think he was in a difficult position too. How does that make you feel?

Patient: I also need to deal with him a lot. It's very tough. You keep thinking I'm bullying him, but you don't understand how he tortures me inwardly.

I started to discern that she was feeling a sense of being attacked by me. To a certain extent, she could be sensible for having that feeling. Using the approach of defining the affected focus in our therapeutic relationship, I attempted to move the implicit process to explicit mentalizing so that the unspoken could be spoken and it was safe to share further.

Therapist: When you said so, I feel that whatever I'm trying to point out to you here, I'm siding with him and neglecting you. Am I right?

Patient: Yup. After so many sessions we had, yes, I have that feeling.

Therapist: It seems like we have different views about something right now. And you sounded pretty upset with me too.

Patient: In our sessions, I feel you are taking a side on him. The very reason is both of you are guys. Imagine you have a girlfriend who's slightly superior to you in many ways, so your partner will feel inferior. If a mature person, he will try to grow up and improvise himself. But in my case, he is not. So, I think most guys don't like their girlfriends to be so aggressive and vocal. They prefer to have a submissive girlfriend so that they can be the hero to protect her.

The patient had this psychic equivalence of non-mentalizing mode that a male therapist would quickly be not siding with her. From a traditional psychoanalytic perspective, the male therapist could represent an object of transference enactment. I now observed a very crucial point that she had talked about here. Although upset with her boyfriend, she looked up to him to protect *her*, the inner sense of a child. However, she struggled to moderate the extent of aggression or submission to him. By being aggressive, on the one hand, she would risk destroying the object that could offer her refuge. By being submissive, on the other hand, she would fear the loss of autonomy and face the resultant self-object, which is deemed vulnerable. In our therapeutic relationship, was she too struggling to moderate the extent to which she should surrender her mind to me (*submissive and weak*) or stay attacking me for not listening to her (*aggressive but wishing for protection*)?

Therapist: Let's pause and rewind a bit to the discussion earlier. I know what you are trying to let me understand here is the feeling that I'm siding with your boyfriend and not acknowledging how difficult it is for you to live with him.

Patient: Yup, I understand that not many guys prefer a girlfriend like me.

The capacity of her thinking had not been eased thus far. Instead, she had reverted to a critical state of mind about herself, as she proclaimed no guy would love a girl like her. The apparent invaluable self-object seemed to take over, including her fear that she was not likable to the therapist.

Therapist: That sounds harsh when you said that to yourself. What runs through your mind when you think I'm siding with him? What makes you have that feeling?

Patient: An example was when I narrated a situation about him, then the advice you give is, have I stand on his side to think and see why he acted that way? It gave me a perception of what I should do then. Do I need to think about others every time in those situations? It's like I'm being blamed for everything that happened. I don't see how this therapy is going to be able to help me. How can I apply this in my life? I remember you told me just keep thinking. So, when I heard of that, it confused me as my mind was already full of many things. Worse, I do not know how to differentiate whose faults. So, instead, I chose to self-blame and dislike myself.

Quite clearly, the client had difficulty thinking about others or even withstanding the thought that “I need to think about others” in her mind. The thinking process in this realm forced her to deal with the gap that her inner needs conflicted with the demand of external reality.

Therapist: I did not know that asking you to consider others' perspectives and feelings is causing you to feel so bad toward yourself.

Patient: Yes, as if no one can understand me. Of course, besides stepping onto their shoes, I'm also making it hard for them to cross mine.

In this sense, the patient talked about how important it was to protect her inner needs, even at the expense of being left alone. So, apart from these, what had caused her such inhibitions of thoughts for others? I reflected that it is a need for protection. The patient elaborated on how she was dumb as she could not derive any improvements or changes from the therapy. She continued to be harsh and critical toward herself. There is a more overt manifestation of the superego harshness. The superego, according to Freud, is the internalized parental figure carrying the parental prohibitions, which becomes a structure in our unconscious mind. This internal authority had forbidden her thought.

## 4. Discussion

This case study illustrated the inhibitions of one's thinking, which resulted in self-harm and suicidal behaviors, can be explained by integrating traditional psychoanalytic and contemporary mentalizing theoretical perspectives based on the hypothesized pathologies of having conflictual mental representations and dysfunctional mental processing.

First, the conflictual mental representations from a traditional psychoanalytic point of view could be seen from the early object relations between the patient and her father and subsequent relationships with the boyfriend and the therapist. There was a fearful superego in the patient's inner world, one which she had become afraid of to question its validity—an internalized harsh father figure who was ever restricting and punishing her search for knowledge and freedom of thought (7). At the same time, the superego may also represent her projections into this father figure of her aggressive impulses and phantasies. Thus, this fearful superego would always be harsh toward herself and others, giving rise to the lens of viewing herself and people as never good enough. By conflict, the superego also demanded it to be treated as the perfect father, who was never exposed to any critical thought. This could be understood as her own need or phantasy for such an ideal father, who had consistently failed her from within when it came to taking refuge from emotional turmoil. So, these internal and external object representations, which stemmed from the superego's core conflicts, had substantially contributed to her difficulties, including suicidal tendencies.

Second, the mentalizing perspective suggests a dysfunctional mental processing in the patient, as she could not mentalize or think about the inner states of herself and others. Her cognitive processing was at a default state of being self-focused, even amounting to some self-aggrandizement. She was susceptible to non-verbal and verbal communication cues and eager to judge how people treated her based on external features and perceptions. She could not slow down this inference process as it was often not subjected to internal scrutiny. Maintaining mentalization in attachment relationships is incredibly challenging for someone with hyperactive attachment systems because of their history and/or biological predisposition (10). The fearful-avoidant attachment pattern, seen in this case, had manifested itself as “reluctant to engage in a close relationship but a dire need to be loved and understood by others” (11). People with this attachment pattern have elevated anxiety in perceiving others negatively and having negative views about themselves. All these difficulties were evident in the patient's ability to subject her thoughts to examination and reality testing, even those involving risk to herself.

To highlight these further, Fonagy and Target (1993) distinguished and described two models of the psychoanalytic treatment of mental disturbance and their associated pathologies (12). The first model deals with helping a client recover threatening ideas and distorted feelings because of conflicting mental representations and defenses. In contrast, the second model involves engaging previously inhibited cognitive processes and dysfunctional mental processing within the psychoanalytic encounter. Almost all forms of psychotherapeutic work aim for change. In an all-inclusive way, these changes could include corrective experiences and new behaviors, a new understanding of oneself, an improvement in the level of hope and expectancy, better therapeutic alliance and relationship, and a promotion in reality testing (13, 14). To change, the precursor is the involvement of thinking. Psychoanalysis, as the most intense psychotherapy, aims for a specific change, not so much on symptom alleviation but on underlying structural change (15).

Nevertheless, structural change, as it said, was classically defined by Rapaport (1957) as “quasi-permanent organizations” (16) and later as “configurations with a slow rate of change” (17). Thus, in these views, psychic structures are relatively stable and permanent; in a way, they resist change. However, the success of psychoanalysis in bringing about deep-seated changes in personality and emotional development has been recognized (18). These demonstrate that structural change is possible, albeit rather slowly. These discoveries certainly brought about a wave of therapeutic optimism. The change process in psychotherapy is influenced by the net effects of one's aptitude for thinking and inhibition.

So, what we would add trying to integrate these two perspectives is, perhaps, the bridging point from which thought and thinking are indeed the outcomes of a complex interaction of our impulses, wishes, phantasies, and perceptions, regardless of how deep down they are within our inner world. We can conclude that we cannot mentalize well because of attachment anxiety and that there are primitive wishes and phantasies which stop us from advancing our thinking. We deal with our most innate needs, whether mentalizing or thinking against the omnipotence phantasy. The realization that “*This is not what it is, it is what I made it to be in my mind; I have been thinking such and such*” marks the beginning of mentalization about others and oneself. An internal conversation like this is about realizing that no matter how omnipotent our fantasies, wishes, or impulses are, they can be converted to a thought, tested, examined, or possibly altered. As Hanna Segal (1977) said, thinking evolves from omnipotent phantasy, a phantasy recognized as one that can be subjected to reality testing (7).

## 5. Conclusion

This case report represents an attempt to integrate the hypothesized inhibitions on one's capability to think, involving one's own core conflicts and dysfunctional mental processing from the traditional psychoanalytic and mentalizing perspectives. The main idea of this theoretical integration is to highlight that psychotherapy can be used to recover threatening thoughts and distorted feelings because of conflicting mental representations, followed by mentalizing previously inhibited or dysfunctional cognitive processing within the psychoanalytic encounter. Further study into these processes and their influence on the suicidal process are essential as it can potentially improve suicide risk assessment and prevention with a more solid understanding of suicidal phenomena, apart from enhancing psychotherapeutic outcomes.

## Data availability statement

The raw data supporting the conclusions of this article will be made available by the authors, without undue reservation.

## Ethics statement

The studies involving human participants were reviewed and approved by the Medical Research and Ethics Committee, Ministry of Health Malaysia. The patients/participants provided their written informed consent to participate in this study. Written informed consent was obtained from the participant/patient(s) for the publication of this case report.

## Author contributions

CS collected the case history and composed the manuscript.
